# Not Just for Ligation: Repurposing the Gastroduodenal Artery as a Vascular Conduit During Pancreaticoduodenectomy

**DOI:** 10.7759/cureus.110176

**Published:** 2026-06-03

**Authors:** Karthik Dhanireddy, Shyam Allamaneni, Jason Batey

**Affiliations:** 1 General Surgery, Bon Secours Mercy Health - The Jewish Hospital, Cincinnati, USA; 2 Surgical Oncology, Bon Secours Mercy Health - The Jewish Hospital, Cincinnati, USA

**Keywords:** bypass, cancer, gda re-implantation, pancreatic cancer, whipple

## Abstract

In a standard pancreaticoduodenectomy (Whipple procedure), the gastroduodenal artery (GDA) is ligated. In a patient with celiac axis occlusion with prominent GDA collaterals, the decision of whether to preserve the GDA comes into play. We report the case of an otherwise healthy 60-year-old woman who presented with a pancreatic mass with concurrent celiac axis occlusion. Pre-operative imaging illustrated a celiac axis occlusion with prominent collaterals from the gastroduodenal artery supplying the distal pancreas, spleen, and stomach. Arteriography further demonstrated a replaced right hepatic artery originating from the superior mesenteric artery and a diminutive left hepatic artery. Based on these findings, the decision was made to re-implant the gastroduodenal artery to preserve the aforementioned structures. Thus far, there have been no instances of GDA re-implantation in the literature. A review of the literature was performed using PubMed, Embase, and Google Scholar with search terms including 'gastroduodenal artery reimplantation,' 'GDA reconstruction,' 'Whipple,' 'pancreaticoduodenectomy,' and 'celiac axis occlusion.' To our knowledge, no prior reports of gastroduodenal artery re-implantation during pancreaticoduodenectomy have been identified.

## Introduction

Celiac axis occlusion is an infrequent event that typically presents as postprandial pain, unintended weight loss, nausea, and vomiting [1]. In select situations, the body adapts to chronic remodeling and occlusion of the celiac artery by promoting the growth of collateral vessels. In these patients, celiac axis occlusion can be asymptomatic. Based on the proximity and distribution of vessels, collaterals can form between any of the superior mesenteric branches and celiac artery branches - often the gastroduodenal artery [2].

In patients with pancreatic cancer undergoing pancreatic head resection with duodenectomy (Whipple procedure), the gastroduodenal artery is the feeding vessel to both these organs and is routinely ligated [3]. In traditional surgical resection of a pancreatic cancer, the vessels taken into consideration are the superior mesenteric artery, celiac axis, common hepatic artery, and the superior mesenteric vein/portal vein confluence [4]. While these vessels play a monumental role in determining resectability, it is also imperative to note other vascular anomalies. Oftentimes, a multi-disciplinary approach with vascular surgery can facilitate reconstruction of vessels either through an interposition graft or re-implantation, while still achieving an R0 resection and ensuring adequate blood flow to adjacent structures [5].

## Case presentation

We present the case of a 60-year-old female with a past medical history notable only for a prior cholecystectomy and appendectomy, who presented in the emergency room at an outside hospital for nausea and vomiting with associated epigastric pain. Labs were significant for elevated transaminases. Cross-sectional imaging showed a questionable pancreatic mass. She subsequently underwent an endoscopic ultrasound, which confirmed a 1.8 cm ill-defined hypodensity in the pancreatic head with associated common bile duct dilatation (Figure 1). This was further biopsied, confirming pancreatic adenocarcinoma. Staging was negative for metastatic disease. 

**Figure 1:**
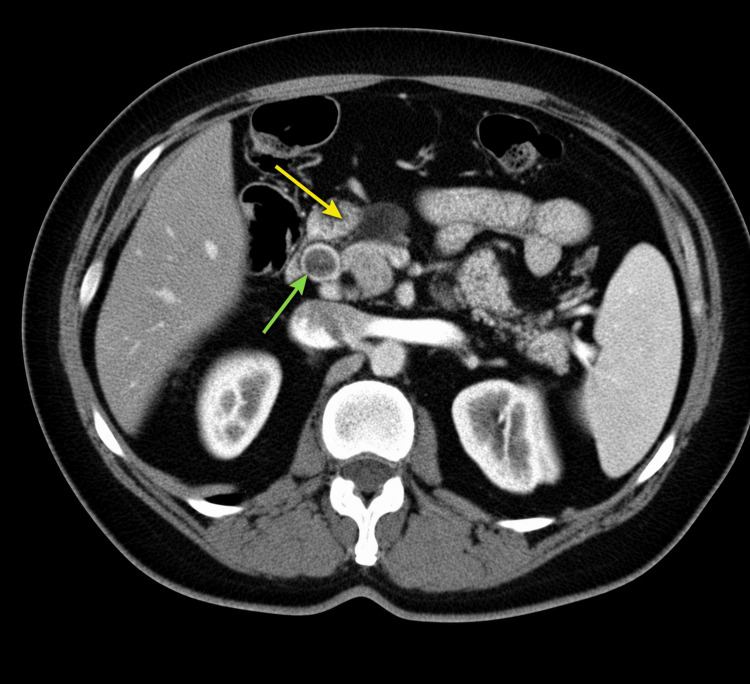
Cross-sectional CT of pancreatic mass with associated common bile duct dilatation Green arrow represents common bile duct dilatation of 10 mm. Yellow arrow represents an ill-defined pancreatic mass.

Aberrant vascular anatomy was noted on the primary surgeon’s review of the CT scan when seen in the office. This was discussed with radiology at the time, who agreed that there was occlusion at the origin of the celiac artery, with a hypertrophied gastroduodenal artery. To further evaluate this, vascular surgery was consulted. The patient underwent formal mesenteric arteriography. Findings were consistent with a diminutive left hepatic artery with GDA collaterals supplying the left lobe of the liver, celiac and proper hepatic artery occlusion (Figure 2), a replaced right hepatic artery coming off the superior mesenteric artery (Figure 3), and a splenic artery supplied solely by the gastroduodenal artery. 

**Figure 2:**
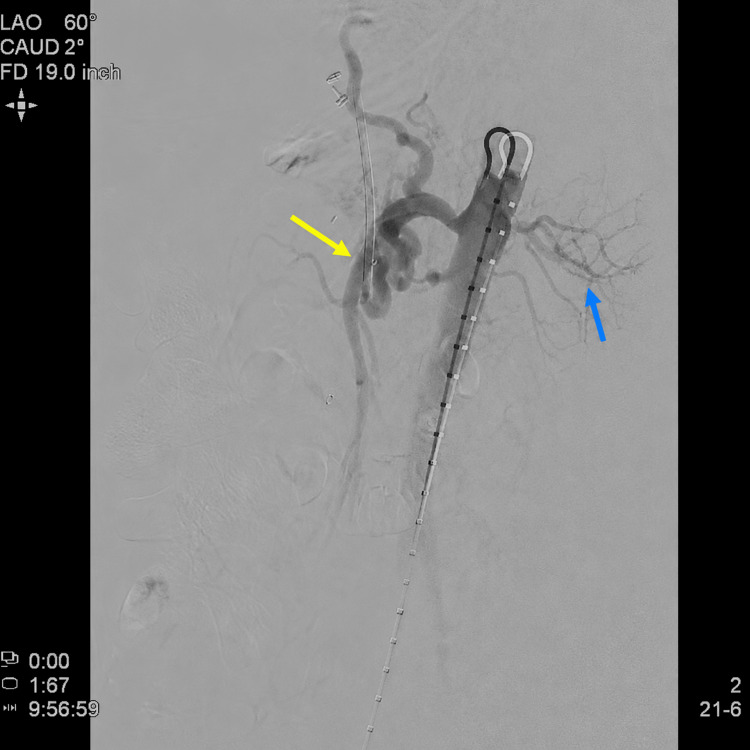
Arteriography revealing celiac axis occlusion with collaterals supplying the spleen Yellow arrow illustrates the celiac axis. Blue arrow points to the splenic collateralization.

**Figure 3:**
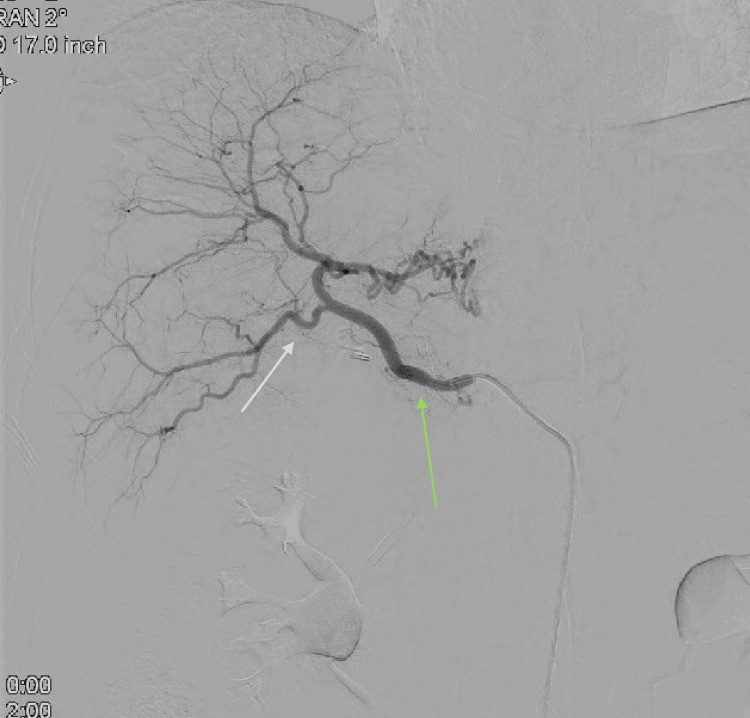
Arteriography showing replaced right hepatic artery coming off of the superior mesenteric artery White arrow points to the replaced right hepatic artery. Green arrow points to the superior mesenteric artery.

Given that the left lobe of the liver, the distal pancreas, and the spleen were all supplied by the GDA and its collaterals, the decision was made to preserve the GDA preoperatively. After all surgical planning was complete, the patient went to the operating room for a pylorus-preserving pancreaticoduodenectomy. After kocherizing the duodenum and taking down attachments in creating the retropancreatic tunnel, attention was turned to the large hypertrophied gastroduodenal artery. The supraceliac aorta was exposed in standard fashion, and the gastroduodenal artery was dissected out until it was able to be reflected nicely to the level of the aorta. The patient was then heparinized at a dose of 100 units/kg. A side-biting clamp was used to allow for continued aortic perfusion downstream, and an end-to-side anastomosis was performed. After re-implantation, there was an excellent pulse in the common hepatic artery and excellent Doppler tones in the previously diminutive left hepatic artery. The liver and spleen were normal in appearance at the conclusion of the case. No protamine was given. The pancreaticoduodenectomy was then subsequently finished with resection of the pancreatic head and duodenum, along with three concomitant anastomoses in the usual fashion. 

Postoperatively, the patient did well and was discharged on hospital day seven on 81 mg of aspirin. Ten years later, she has had no recurrence of her symptoms, no need for repeat intervention, and continues to live cancer-free. Repeat cross-sectional imaging at the one-, five-, and ten-year follow-ups showed widely patent vessels (Figure 4).

**Figure 4:**
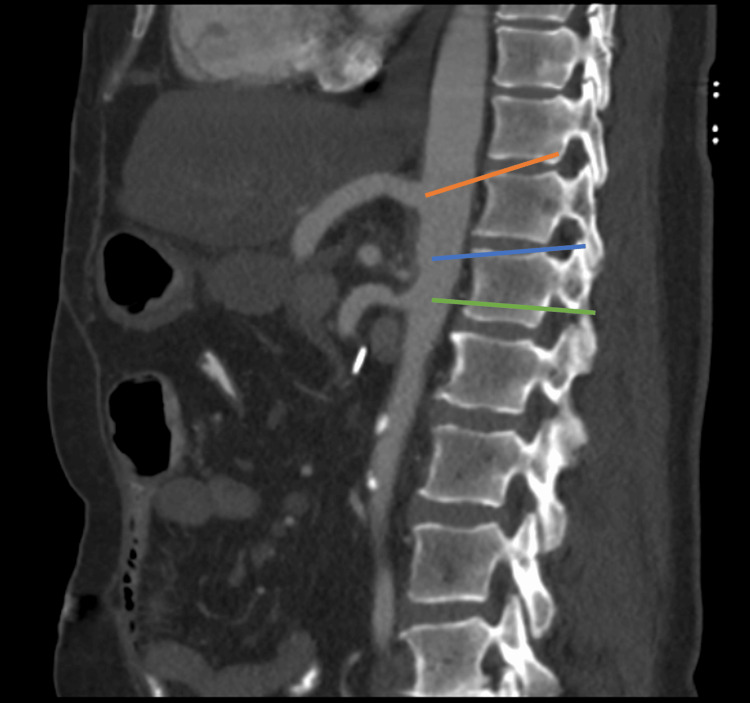
Repeat cross-sectional imaging post-procedure ten years later Orange - implanted gastroduodenal artery. Blue - celiax axis occlusion. Green - superior mesenteric artery.

## Discussion

Concomitant vascular reconstruction during pancreatic surgery is not uncommon, given the close proximity of major vascular structures and their associated collateral networks. A multi-institutional retrospective review published in 2023 demonstrated that up to 23% of patients undergoing pancreaticoduodenectomy required some form of vascular reconstruction. Despite the increased technical complexity, 30-day survival in this cohort was 93%, compared to 96% among patients who did not require reconstruction [6]. These favorable short-term outcomes highlight the critical role of meticulous preoperative planning and the availability of multidisciplinary expertise.

Preoperative planning remains a cornerstone of safe surgical practice. While it typically involves a comprehensive assessment of the patient’s overall health, comorbidities, and operative risk, it also necessitates careful review of preoperative imaging [7]. This case underscores the importance of attention to subtle but clinically significant findings. On the initial radiologic interpretation, the celiac axis occlusion was not identified, and there was no mention of a hypertrophied gastroduodenal artery (GDA). Failure to recognize this anatomy could have had significant consequences, as routine ligation of the GDA would have eliminated the dominant collateral pathway perfusing the foregut organs distal to the celiac occlusion. This could have resulted in compromised perfusion of the stomach and spleen, with potential hepatic ischemia depending on the adequacy of collateral and variant arterial supply.

Although our institution is classified as a community hospital, it offers a broad range of surgical expertise, including complex surgical oncology, advanced vascular surgery, and cardiothoracic surgery. While outcomes for complex pancreatic and vascular procedures have generally been shown to be superior at high-volume tertiary care centers [8], successful management of highly specialized cases remains dependent on institutional expertise, multidisciplinary collaboration, and the availability of appropriate operative and perioperative resources. In carefully selected patients, centers possessing these capabilities may achieve safe and effective outcomes comparable to those reported by larger referral institutions.

In this case, the adequate length of the gastroduodenal artery allowed for successful transposition to the supra-celiac aorta. However, contingency planning was essential. Both lower extremities were prepped in anticipation of a potential interposition graft if direct transposition proved unfeasible. The preferred alternative would have been an aorto-GDA interposition using the greater saphenous vein. However, given the patient's favorable vascular anatomy and the ability to achieve a tension-free anastomosis with adequate vessel length and caliber, direct GDA transposition was favored to avoid the additional operative time and potential graft-related complications associated with vein harvest and interposition grafting. Although synthetic conduits such as polytetrafluoroethylene (PTFE) or polyethylene terephthalate are viable options, evidence from the broader vascular surgery literature generally demonstrates superior long-term patency and resistance to infection with autologous venous conduits compared with prosthetic grafts. While data specific to hepatopancreatobiliary arterial reconstruction are limited, these principles often inform conduit selection in complex visceral vascular reconstructions. [9]. This advantage is largely attributable to their inherent biocompatibility, which confers improved compliance and reduced susceptibility to infection [10].

## Conclusions

This case highlights the critical importance of meticulous preoperative evaluation and individualized operative planning in pancreatic surgery, particularly in the presence of aberrant vascular anatomy. Recognition of a previously unreported celiac axis occlusion with compensatory hypertrophy of the gastroduodenal artery was essential in preventing catastrophic hepatic and splenic ischemia. Successful revascularization via GDA transposition to the supraceliac aorta enabled safe completion of pancreaticoduodenectomy with preservation of end-organ perfusion.

Furthermore, this case underscores that complex vascular reconstructions can be performed safely outside of traditional high-volume tertiary centers when appropriate expertise, multidisciplinary collaboration, and contingency planning are in place. The patient’s excellent long-term outcome, with durable vascular patency and sustained oncologic control at 10 years, reinforces the feasibility and durability of such an approach.

Ultimately, careful attention to anatomic detail, thoughtful surgical planning, and adaptability in the operating room remain paramount to achieving optimal outcomes in complex hepatopancreatobiliary surgery. Although conclusions are limited by the single-patient nature of this report, the favorable long-term vascular and oncologic outcomes observed suggest that gastroduodenal artery re-implantation may represent a viable reconstructive strategy in select patients with complex visceral arterial anatomy.
